# Chemotherapy tolerance and continuity are prognostic for overall survival in patients with localized and advanced biliary tract cancer

**DOI:** 10.55730/1300-0144.6102

**Published:** 2025-10-06

**Authors:** Erman AKKUŞ, Pınar KUBİLAY TOLUNAY, Elif Berna KÖKSOY, Hatime Arzu YAŞAR

**Affiliations:** 1 Division of Medical Oncology, Department of Internal Medicine, Faculty of Medicine, Ankara University, Ankara, Turkiye; 2 Cancer Research Institute, Ankara University, Ankara, Turkiye

**Keywords:** Biliary tract cancer, chemotherapy, eligibility, tolerance, survival, real-world

## Abstract

**Background/aim:**

Biliary tract cancers (BTC) are relatively rare and have a poor prognosis in both localized and metastatic settings. Clinical trials tend to include patients who can tolerate treatments; however, chemotherapy eligibility, patterns, and survival may differ in the real world. The present study provides a 5-year overview of chemotherapy eligibility, patterns, tolerance, and survival in patients with resected and advanced BTCs.

**Materials and methods:**

Included in the study were patients with resectable or advanced BTC (excluding ampullary cancers) diagnosed between 2019 and 2024. The demographic/clinical characteristics, chemotherapy eligibility, patterns, and survival outcomes of the patients were evaluated.

**Results:**

Of the 151 patients included in the study, 61 (40.7%) had resected BTC and 90 (59.3%) had advanced BTC. Among the patients with resected BTC, only 52.5% received adjuvant chemotherapy, 38.7% needed dose reductions, and 29% could not complete the planned cycles. Median recurrence-free survival and overall survival (OS) were 24.1 months (95% confidence interval (CI): 11.4–58.0) and 59 months (95% CI: 38.4–59) in patients with resected BTC, respectively, for all patients. In a multivariable analysis, only the number of adjuvant chemotherapy cycles was associated with OS [Hazard ratio (HR):0.63 (95% CI: 0.39–1.00), p=0.050]. Among the patients with advanced disease, 16.7% were not eligible for first-line chemotherapy, and 70.7% needed dose reduction. The median number of cycles was three (0–18); grade 3–4 adverse events were observed in 52% of the patients; and median progression-free survival and OS were 4.3 months (95% CI: 3.3–5.0) and 9.4 months (95% CI: 5.9–13.7) for all patients, respectively. Only 36.7% were able to receive second-line treatment. The number of first-line chemotherapy cycles [HR: 0.58 (95% CI: 0.45–0.76), p < 0.001]/discontinuation due to toxicity [HR: 3.26 (95% CI: 1.34–7.93), p = 0.009], cisplatin-gemcitabine regimen [HR: 0.10 (95% CI: 0.01–0.58), p<0.001], and receiving second-line chemotherapy [HR: 0.28 (95% CI: 0.11–0.68), p < 0.001] were significantly associated with OS in multivariable analyses.

**Conclusion:**

This study shows that a significant proportion of patients with BTC are not eligible or intolerant to chemotherapy in the real world. Maintaining the planned treatment, even with dose reduction, is associated with better OS.

## 1. Introduction

Biliary tract cancers (BTC), including intrahepatic, perihilar, distal cholangiocarcinomas, and gallbladder cancers, are relatively rare, accounting for less than 3% of all gastrointestinal malignancies and 10–15% of primary liver cancers [1]. Adjuvant capecitabine after resection in localized disease, and first-line cisplatin-gemcitabine combined with immunotherapy in advanced cases are the recommended treatments [2]. However, survival remains poor in both localized and advanced cases. The recurrence rate reaches approximately 80% after resection, and the median overall survival (OS) is 11–13 months, even with the implementation of immunotherapy [2, 3].

Clinical trials tend to include patients who are fit, lack any significant comorbidities, and are able to tolerate chemotherapy, while patients with BTCs may be vulnerable in routine clinical practice. The median diagnosis age in BTCs is approximately 70 years [4]. Biliary surgery is associated with a relatively high frequency of morbidity; and long-term postoperative complications may occur [5, 6]. In one clinical trial, some 40% of the patients were unable to complete planned adjuvant capecitabine therapy [7]. In a clinical trial including advanced cases, only 30% of the patients had an Eastern Cooperative Oncology Group (ECOG) performance score (PS) of 0, and more than 25% of the patients could not complete the first three cycles of cisplatin-gemcitabine [8].

Immunotherapy and targeted agents are reshaping the treatment algorithm of BTCs; however, immunotherapy still offers only modest survival benefits, and novel drugs are still not available or accessible in many countries. Accordingly, chemotherapy remains the primary treatment approach for both localized and advanced BTC. In recognition of the fragility of this patient population indicated in clinical trials, and our observed vulnerability of patients with BTCs in daily practice, we present here a 5-year overview of patients treated at a tertiary center, examining chemotherapy eligibility / patterns, tolerance, and survival in cases with resected and advanced BTCs.

## 2. Materials and methods

### 2.1. Patients and data

This study utilizes retrospective cohort data from a tertiary cancer center in Türkiye. Included in the study were patients of both sexes, aged 18 years or older, with histologically confirmed, resectable, or advanced biliary tract adenocarcinoma, diagnosed between 2019 and 2024. Patients with intrahepatic, perihilar, and distal cholangiocarcinomas and gallbladder cancers were included, whereas those with ampullary cancers were excluded, along with those whose conditions had not been histologically confirmed. All data were retrieved from the Avicenna Hospital Data Management System.

For patients with resected BTC, the following data were recorded: sex, age, ECOG PS, comorbidities (hypertension, diabetes, cardiac disease, hypothyroidism, viral hepatitis, cirrhosis), smoking and alcohol history, tumor localization (intrahepatic, perihilar, distal, and gallbladder), maximum tumor diameter, pT stage, pN stage (positive and negative), resection margin (R0, R1), grade (1,2,3), presence of lymphovascular invasion (LVI) and perineural invasion (PNI), carbohydrate antigen 19-9 (Ca19-9) and carcinoembryonic antigen (CEA) levels at diagnosis, adjuvant chemotherapy types, reasons for not receiving adjuvant chemotherapy, completeness of planned adjuvant chemotherapy, presence of dose reduction, grade 3–4 adverse events, and survival status.

For patients with advanced BTC, the following data were recorded: sex, age, ECOG PS, comorbidities (hypertension, diabetes, cardiac disease, hypothyroidism, viral hepatitis, cirrhosis), smoking and alcohol history, primary tumor localization (intrahepatic, perihilar, distal, and gallbladder), presentation (initially advanced or recurring after resection), metastatic sites, molecular testing, Ca19-9 and CEA levels at diagnosis, first-line chemotherapy type and cycle, dose reduction, grade 3–4 adverse events, response, second-line chemotherapy, and survival status.

Baseline characteristics, chemotherapy eligibility and treatment patterns, and tolerance were recorded, along with recurrence-free survival (RFS) and OS for patients with resected disease. RFS was defined as the time from surgery to the first recurrence of the disease or death, whereas OS was defined as the time from surgery to death. Also recorded were objective response rate (ORR, defined as the proportion of patients with a complete or partial response to treatment according to RECIST 1.1) and progression-free survival (PFS, defined as the time from the initiation of first-line chemotherapy [and pathological diagnosis for patients who did not receive treatment] to initial disease progression or death.). OS was analyzed using univariable and multivariable methods, in patients receiving adjuvant treatment and those receiving first-line chemotherapy, respectively.

Each case was staged based on the American Joint Commission on Cancer (AJCC) TNM system, 8^th^ Edition; adverse event grading was based on Common Terminology Criteria for Adverse Events (CTCAE) Version 5.0; and response to first-line treatment was evaluated according to RECIST 1.1 (Response Evaluation Criteria in Solid Tumors).

Ethical approval was obtained from the Clinical Research Ethics Committee of Ankara University Faculty of Medicine (Number: 655-i09–714–24), and the study was conducted in accordance with the principles of the Declaration of Helsinki. As the study involved the retrospective analysis of anonymous clinical patient data, the requirement for informed consent was waived by the Ethics Committee.

### 2.2. Statistical analysis

Continuous variables were presented as median [minimum (min)–maximum (max)], and categorical variables as percentages. Survival was estimated using the Kaplan-Meier method and compared with the log-rank test. Univariable and multivariable analyses were performed using Cox regression. Variables with a p value of < 0.20 in the univariable analysis were included in the multivariable analysis. All p values were based on a 2-tailed test of significance (p = 0.05). Statistical analyses were conducted using MedCalc^®^ Statistical Software, version 22.026 (MedCalc Software Ltd, Ostend, Belgium).

## 3. Results

### 3.1. Patients with resected BTC

#### 3.1.1. Characteristics and chemotherapy patterns

The baseline characteristics and adjuvant chemotherapy eligibility, patterns, and tolerance of patients with resected BTC are presented in Tables 1 and 2. Of the 61 patients with resected BTC, 57.4% (n=35) were male and the median age at diagnosis was 63 years (41–78); 23% (n=14) and 50.8% (n=31) had ECOG PS 0 and 1, respectively; 36.1% (n=22) had diabetes mellitus, 23% (n=14) had cardiac disease, and 47.5% (n=29) had a smoking history. Gallbladder and intrahepatic cancers were the most prevalent forms (34.4% and 29.5%), while the lymph node positivity, R1 resection, LVI, and PNI rates were 21.3%, 34.4%, 52.5%, and 39.3%, respectively. The median Ca19-9 level was 31.2 U/mL (0.8–6757.0).

Among the treatments provided, 52.5% (n=31) of the patients received adjuvant chemotherapy, with gemcitabine-capecitabine (24.6%, n=15) and capecitabine (14.8%, n=9) being the most common regimens. Furthermore, 38.7% of the patients required dose reductions, and 29% were unable to complete the planned adjuvant chemotherapy cycles due to toxicity, as presented in Table 2. Grade 3–4 adverse events were observed in 29% of the patients, and postoperative complications were the most common reason for not being provided adjuvant chemotherapy, occurring in 13.1% of the total.

#### 3.1.2. Survival

**Figure 1:**
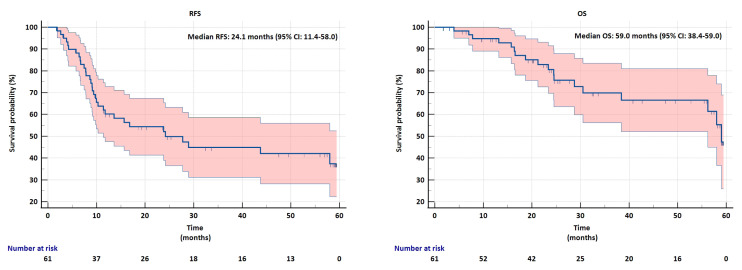
a) Recurrence-free survival (RFS) and b) Overall survival (OS) in patients with resected biliary tract cancer.

The median follow-up time was 24.6 months (min–max:1.7–59.4). The median RFS for all patients with resected BTC was 24.1 months (95% CI: 11.4–58.0) (Figure 1a), and the median OS was 59 months (95% CI: 38.4–59.0) (Figure 1b).

**Figure 2:**
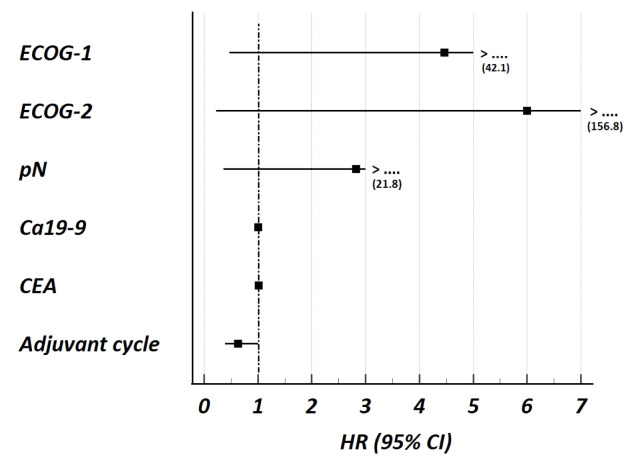
Forest plot presentation of multivariable Cox regression analysis for overall survival (OS) in patients who received adjuvant chemotherapy for resected biliary tract cancer. Only the adjuvant chemotherapy cycle was statistically significant, revealing that a higher number of chemotherapy cycles (continuing and completeness) was associated with better OS (Univariable analyses and complete HR (95% CI) presented in Table 3). Abbreviations: ECOG: Eastern Cooperative Oncology Group, Ca19-9: carbohydrate antigen 19 9, CEA: carcinoembryonic antigen, HR: Hazard ratio, CI: Confidence interval.

Among the patients started on adjuvant chemotherapy, the median OS was 59 months (95% CI: 16.1–101.8). Patient characteristics were analyzed to identify any association with OS in those receiving adjuvant chemotherapy. The univariable and multivariable analyses are presented in Table 3, and the results of the multivariable analyses are presented in Figure 2. ECOG PS, pN positivity, CEA, and Ca19-9 levels, and the number of adjuvant chemotherapy cycles received were included in the multivariable analysis, which was based on the findings of the univariable analyses. Only the number of adjuvant chemotherapy cycles was found to be significantly associated with OS [Hazard ratio (HR): 0.63 (95% CI:0.39–1.00), p = 0.050], suggesting that a higher number of cycles is associated with better OS.

### 3.2. Patients with advanced BTC

#### 3.2.1. Characteristics and chemotherapy patterns

Tables 4 and 5 present the baseline characteristics and first-line chemotherapy eligibility, patterns, and tolerance in patients with advanced BTC. Included in the study were 90 patients with advanced BTC, of whom 54.4% (n=49) were male with a median age at diagnosis of 67 years (39–89). Among the patients, 14.4% (n=13) and 54.4% (n=49) had ECOG PS 0 and 1, respectively; 36.7% (n=33) had diabetes mellitus, 18.9% (n=17) had cardiac disease, and 44.4% (n=40) had a history of smoking. Intrahepatic cancers and gallbladder primaries accounted for the majority of cases (51.1% and 17.8%, respectively); 31.1% (n=28) developed recurrent disease after the resection of localized disease, and of these, 50% had previously received adjuvant chemotherapy. Furthermore, 84.4% (n=76) of the patients had liver metastases, and 73.3% and 67.8% did not undergo next-generation sequencing (NGS) testing for molecular targets or microsatellite instability (MSI) testing. The median Ca19-9 level at the time of diagnosis was 105.0 U/mL (min–max: 0.8–19960.0).

Some 83.3% (n=75) of the patients were eligible for first-line chemotherapy, with cisplatin-gemcitabine being the most common regimen. Among the patients who received first-line chemotherapy, 70.7% required dose reduction. Grade 3–4 adverse events were observed in 52% of the patients and 28% discontinued the treatment due to toxicity before completing the planned cycles. The median cycle of first-line treatment was 3 (min–max: 0–18). ORR to the first-line chemotherapy was 29.3%. Among all patients, 36.7% were able to receive second-line treatment, while none received a targeted treatment.

#### 3.2.2. Survival

**Figure 3:**
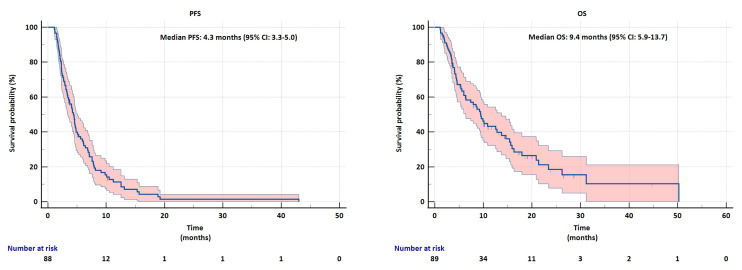
a) Progression-free survival (PFS) and b) Overall survival (OS) in patients with advanced biliary tract cancer.

The median follow-up time was 7.6 months (min–max:1.1–50.2). While the median PFS for all patients with advanced BTC was 4.3 months (95% CI: 3.3–5.0) (Figure 3a), the median OS was 9.4 months (95% CI: 5.9–13.7) (Figure 3b).

**Figure 4:**
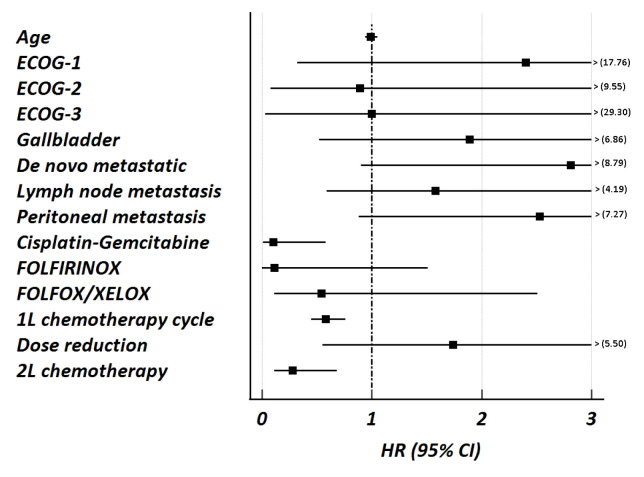
Forest plot presentation of multivariable Cox regression analysis for overall survival (OS) in patients who received first-line chemotherapy for advanced biliary tract cancer. Cisplatin-gemcitabine, number of 1L chemotherapy cycles, and receiving second-line chemotherapy were significantly associated with better OS (Univariable analyses and complete HR (95% CI) presented in Table 6). Abbreviations ECOG: Eastern Cooperative Oncology Group; FOLFIRINOX: fluorouracil, leucovorin, irinotecan, and oxaliplatin; FOLFOX: fluorouracil, leucovorin, and oxaliplatin; XELOX: capecitabine and oxaliplatin, 1L: first-line, 2L: second-line, HR: Hazard ratio, CI: Confidence interval.

Among the patients who started first-line chemotherapy, the median OS was 10.8 months (95% CI: 7.3–14.4). Characteristics associated with OS in patients receiving first-line chemotherapy were analyzed. The univariable and multivariable analyses are presented in Table 6, and the results of the multivariable analyses are presented in Figure 4. Age, ECOG PS, primary localization, initially advanced disease, lymph node and peritoneal metastasis, type of first-line chemotherapy, number of cycles, dose reduction, and receiving second-line treatment were included in the multivariable analysis, which was based on the findings of the univariable analyses. The number of first-line chemotherapy cycles [HR: 0.58 (95% CI: 0.45–0.76), p < 0.001], cisplatin-gemcitabine regimen [HR: 0.10 (95% CI: 0.01–0.58), p < 0.001] and receiving second-line chemotherapy [HR: 0.28 (95% CI: 0.11–0.68), p < 0.001] were significantly associated with OS.

As discontinuation of treatment due to early disease progression (< 2 months) can confound the results, an additional sensitivity analysis was performed to identify discontinuations resulting from toxicity, excluding patients who had early progression (< 2 months) and who discontinued treatment as a consequence (Table 7). The multivariable analysis revealed treatment discontinuation due to toxicity to be associated with worse OS [HR: 3.26 (95% CI: 1.34–7.93), p=0.009].

## 4. Discussion

This study has presented a 5-year real-life overview of the chemotherapy patterns and survival of cases with resected and advanced BTCs treated in a tertiary oncology center in a developing country. The results indicate that patients with BTC are a fragile population, with treatment patterns and outcomes that deviate from those recorded in clinical trials. The ability of a patient to continue treatment is significantly associated with OS in both localized and advanced stages, even when dose reductions are required.

Although the incidence and mortality associated with BTCs varies considerably worldwide, both have been found to increase substantially, by 76% and 65%, respectively, from 1990 to 2017 [9]. Incidence peaks between the ages of 65–69 years in males and 75–79 years in females [9]. In the present study, patients with resected BTC had a median age of 63, while those with advanced disease had a median age of 67. In the BILCAP adjuvant capecitabine trial, the median age was 62 years in the capecitabine arm and 64 years in the observation arm, similar to the present study [7]. However, in a first-line cisplatin-gemcitabine trial involving advanced stage cases (ABC-02), the median age was 63 years [8], which is lower than in our study. Although the median age in the early stage seems to be lower than in the advanced stage in our study, the median ages are close to those of other common gastrointestinal malignancies, such as 72.5 years for colorectal cancer [10] and 67 years for gastric cancer [11]. In line with the median age, patients with BTCs also have a comorbidity burden. In the French population of BTC, nearly 30% of the patients had a Charlson Comorbidity Index score of 2 or more [12]. In the present study, 23% of patients in the resected group and 18.3% of those in the advanced group had a cardiac comorbidity. Clinical trials generally exclude patients with significant comorbidities, and do not report detailed comorbidities, leading to difficulties in the implementation and interpretation of treatments in daily practice.

Several risk factors have been linked to BTCs, although most patients have no specific etiology. Case-control and cohort studies have reported diabetes mellitus to be a risk factor for both intrahepatic and extrahepatic cholangiocarcinoma [13]. Consistent with these results, 36.1% and 36.7% of the patients in the present study with resected and advanced disease had diabetes mellitus. Almost half of the patients in our study had a history of smoking, which has been reported to be a risk factor for BTCs in an earlier study [14].

In the BILCAP trial, 45% of the patients had an ECOG PS of 0, compared to 32.4% in the ABC-02 trial [7, 8]. In contrast, only 23% and 14.4% of the patients had an ECOG PS of 0 in the present study. PS and comorbidities affect chemotherapy preferences as well as survival in daily practice, leading to deviations from the standard recommendations.

In the BILCAP study, the R1 resection rate was 38% [7]. This is similar to the rate of 34.4% identified in the present study. These high R1 resection rates reflect the complex nature of biliary surgery, and explain the high frequency of postoperative complications precluding adjuvant chemotherapy. In the BILCAP trial, 55% of the patients were able to complete eight cycles of capecitabine, and 46% required dose reduction [7]. In contrast, in the present study, conducted in a working clinic, almost half of the patients did not even start adjuvant chemotherapy, due primarily to postoperative complications. Among those who started adjuvant treatment, 38.7% required dose reduction, and 29% could not complete planned cycles due to toxicity. Treatment discontinuation, reflected in the number of chemotherapy cycles received, was associated with worse OS in multivariable analyses. Despite all these factors, the median RFS in the present study was similar to that recorded in capecitabine arm of the BILCAP study (24.1 vs 24.4 months) [7]. In the ASCOT trial, investigating S-1 as an adjuvant treatment in Japanese patients, an RFS of 3.5 years was reported, which may indicate heterogeneity between clinical trials and the outcomes in clinical practice, as well as possible differences among ethnicities [15, 16].

In the advanced group in the present study, 16.7% of the patients were initially not eligible for treatment. Dose reduction was required in 70% of those who started first-line chemotherapy, and the median cycle number was only three. Almost half of the patients experienced grade 3 or 4 toxicity. In the ABC-02 trial, on the other hand, 73.5% received at least four cycles of cisplatin-gemcitabine, and in the first 12 weeks of treatment, an average of 95% of the planned dose was delivered to patients [8]. Our results, recorded in a working practice, differ from those of controlled clinical trials, suggesting the ineligibility or intolerance of patients to chemotherapy. PFS and OS with cisplatin-gemcitabine in the ABC-02 trial were 8 months and 11.7 months, respectively, while the median OS for all patients in our cohort was 9.4 months, and 10.8 months for those who started first-line chemotherapy, suggesting the vulnerability of patients treated in working clinics. All the mentioned factors contribute to the significant deviations in treatment patterns from the guideline recommendations, resulting in heterogeneity in clinical practice, and limiting the available treatment options.

Options for the treatment of BTCs are rapidly evolving with the advances in immunotherapies and targeted agents. Adding immunotherapy to chemotherapy in the advanced disease setting provides modest benefits [3, 17]. In the KEYNOTE-966 and TOPAZ-1 trials, the addition of pembrolizumab or durvalumab to the cisplatin-gemcitabine protocol contributed only 1.8 and 1.6 months survival gain, with median OSs of 12.7 and 12.9 months, respectively [3, 18]. Interestingly, a study involving 666 patients reporting global survival figures following treatment with durvalumab plus gemcitabine and cisplatin in clinical practice reported a median OS of 15.1 months, exceeding that of clinical trials [19]. However, this study included only patients who could complete the treatment, and did not report any treatment discontinuations other than those resulting from progression or death, the median cycle number, or any dose reductions. It would appear from the “Methods” section that all patients completed their chemotherapy cycles unless their disease progressed.

Targeted agents, when predictive mutations such as NGS are detected in molecular testing, provide significant benefits in advanced BTCs. For example, pemigatinib, a fibroblast growth factor receptor (FGFR) inhibitor, provides a median OS of 17.5 months as a second-line treatment [20]. Currently, targeted therapies related to *FGFR2, IDH1, HER2, NTRK, BRAF, RET, BRCA 1/2, PALB2*, and microsatellite instability are recommended after the first-line treatment if suitable alterations are detected [21, 22]. However, immunotherapy and novel agents are not reimbursed in most countries, especially in the low- and middle-income nations, where chemotherapy remains the core treatment. For this reason, our study did not include patients who were treated with targeted agents. Furthermore, only 26.7% of the patients underwent NGS testing, as although NGS testing is available for patients in our country, the lack of accessibility to novel treatments limits its ordering and use.

In patients with localized disease, multivariable analysis indicated that only the number of cycles was associated with OS. In contrast, among patients with advanced disease, the number of first-line chemotherapy cycles was significantly associated with OS. Furthermore, access to second-line treatment was also significantly associated with OS. These results suggest that treatments should be managed in daily practice to ensure continuation, including starting with lower doses, reducing doses as needed, managing toxicities effectively, and providing effective supportive care. Our results support the cisplatin-gemcitabine regimen as the first-line preferred regimen in BTCs.

Our study has several limitations, primarily its retrospective design, and the small number of cases in the resected group. Multicenter and multicountry studies would contribute to a more accurate overview. Furthermore, the study lacked comprehensive geriatric assessment results, and the treatment regimens were highly heterogeneous, which can be attributed to the heterogeneity and complexity of the patients treated in a clinical practice and the lack of options with high benefits. Immunotherapy and targeted agents are not reimbursed in our country, further limiting the results, and chemotherapy is still the main component of the treatment algorithm. The results of the present study may shed light on future combinatory regimens.

In conclusion, this study has shown that a significant proportion of BTC cases are not eligible for, or intolerant to chemotherapy. Receiving a higher number of chemotherapy cycles is associated with better OS. Furthermore, continuing the planned treatment, even with dose reductions if required, is associated with better survival in both the early and advanced stages. The provision of effective palliative care, tolerable and effective treatment options, access to molecular testing, and novel targeted treatments and immunotherapy are crucial, especially for low- and middle-income countries.

## Figures and Tables

**Table 1:** Characteristics of patients with resected biliary tract cancer.

*Variable*	*N=61*

**Sex, n (%)**	
**Male**	**35 (57.4)**
**Female**	**26 (42.6)**

**Age at diagnosis, median (min-max)**	**63 (41–78)**

**ECOG PS, n (%)**	
**0**	**14 (23)**
**1**	**31 (50.8)**
**2**	**8 (13.1)**
**3**	**4 (6.6)**
**Unknown**	**4 (6.6)**

**Comorbidities, n (%)**	
**Hypertension**	**34 (55.7)**
**Diabetes mellitus**	**22 (36.1)**
**Cardiac disease**	**14 (23)**
**Hypothyroidism**	**9 (14.8)**
**Viral hepatitis**	**5 (8.2)**
**Cirrhosis**	**1 (1.6)**

**Smoking history, n (%)**	**29 (47.5)**

**Regular alcohol consumption, n (%)**	**5 (8.2)**

**Tumor localization, n (%)**	
**Intrahepatic**	**18 (29.5)**
**Perihilar**	**12 (19.7)**
**Distal**	**10 (16.4)**
**Gallbladder**	**21 (34.4)**

**Maximum tumor diameter, cm, median (min-max)**	**4 (1–15)**

**pT stage, n (%)**	
**1**	**17 (27.9)**
**2**	**24 (39.3)**
**3**	**17 (27.9)**
**4**	**2 (3.3)**
**Unknown**	**1 (1.6)**

**pN stage, n (%)**	
**Positive**	**13 (21.3)**
**Negative**	**48 (78.7)**

**Resection, n (%)**	
**R1**	**21 (34.4)**
**R0**	**39 (63.9)**

**Grade, n (%)**	
**1**	**10 (16.4)**
**2**	**40 (65.6)**
**3**	**9 (14.8)**
**Unknown**	**2 (3.2)**

**LVI, n (%)**	
**Positive**	**32 (52.5)**
**Negative**	**9 (14.8)**
**Not reported**	**20 (32.7)**

**PNI, n (%)**	
**Positive**	**24 (39.3)**
**Negative**	**9 (14.8)**
**Unknown**	**28 (45.9)**

**Ca19-9, U/mL, median (min-max)**	**31.2 (0.8–6757.0)**

**CEA, ng/mL, median (min-max)**	**2.2 (0.3–89.5)**

**Abbreviations**: ECOG PS: Eastern Cooperative Oncology Group performance score; LVI: lymphovascular invasion; PNI: perineural invasion, Ca19-9: carbohydrate antigen 19-9, CEA: carcinoembryonic antigen.

**Table 2:** Adjuvant chemotherapy eligibility, patterns, and tolerance in patients with resected biliary tract cancer.

*Variable*	*n=61*

**Adjuvant chemotherapy received, n (%)**	**31/61 (52.5)**
**Capecitabine**	**9 (14.8)**
**Gemcitabine-capecitabine**	**15 (24.6)**
**Cisplatin-gemcitabine**	**3 (4.9)**
**Gemcitabine**	**3 (4.9)**
**XELOX**	**1 (1.6)**
**Only radiotherapy**	**1 (1.6)**

**Adjuvant chemotherapy cycle, median (min-max)**	**6 (1–8)**

**Not able to complete planned and started adjuvant chemotherapy, n (%)**	**9/31 (29)**

**Dose reduction required, n (%)**	**12/31 (38.7)**

**Grade 3/4 adverse events, n (%)**	**9/31 (29)**
Myelosuppression-cytopenia (n=4)	
Infection (n=2)	
Nausea-vomiting (n=2)	
Pulmonary embolism (n=2)	
Deep-vein thrombosis (n=3)	
Hand-foot syndrome (n=1)	
Hypersensitivity reaction (n=1)	
Fatigue (n=1)	

**Adjuvant radiotherapy received, n (%)**	**9 (14.8)**

**Adjuvant chemotherapy ** ***not*** ** received, n (%)**	**29/61 (47.5)**
**Postoperative complication**	**8 (13.1)**
**Low ECOG PS**	**4 (6.6)**
**Comorbidities**	**4 (6.6)**
**No patient consent/lost follow-up**	**7 (11.5)**
**T1 or incidental**	**6 (9.8)**

**Abbreviations**: XELOX: capecitabine and oxaliplatin; ECOG PS: Eastern Cooperative Oncology Group performance score.

**Table 3:** Univariable and multivariable Cox regression analysis for overall survival (OS) in patients with resected biliary tract cancer who received adjuvant chemotherapy.

	Univariable		Multivariable	
	
Variable	HR (95% CI)	p	HR (95% CI)	p

**Sex (male vs. female)**	1.12 (0.31–4.01)	0.859	-	-

**Age (continuous variable)**	0.99 (0.91–1.09)	0.979	-	-

**ECOG PS**				
**1 vs 0**	6.67 (0.82–54.10)	**0.076**	4.46 (0.47–42.16)	0.192
**2 vs 0**	14.40 (0.79–259.85)	**0.071**	6.00 (0.23–156.83)	0.282

**Smoking history (present vs absent)**	1.46 (0.41–5.22)	0.553	-	-

**Comorbidities (present vs. absent)**				
**Hypertension**	0.64 (0.17–2.34)	0.501	-	-
**Diabetes mellitus**	0.56 (0.11–2.69)	0.469		
**Cardiac disease**	1.34 (0.34–5.22)	0.669		
**Hypothyroidism**	1.02 (0.11–8.96)	0.892		
**Viral hepatitis**	1.16 (0.14–9.38)	0.886		

**Alcohol use (present vs absent)**	1.02 (0.21–4.84)	0.977	-	-

**Localization**			-	-
**Perihilar vs intrahepatic**	0.26 (0.02–2.39)	0.237		
**Distal vs intrahepatic**	1.42 (0.31–6.46)	0.643		
**Gallbladder vs intrahepatic**	0.66 (0.12–3.65)	0.638		

**T stage**			-	-
**2 vs 1**	1.23 (0.12–11.90)	0.855		
**3 vs 1**	1.77 (0.19–16.13)	0.612		
**4 vs 1**	3.09 (0.17–53.71)	0.437		

**pN (positive vs negative)**	3.65 (0.95–14.04)	**0.059**	2.82 (0.36–21.85)	0.320

**Resection (R1 vs. R0)**	2.01 (0.53–7.53)	0.298		

**Grade**			-	-
**2 vs 1**	2.22 (0.27–17.83)	0.453		
**3 vs 1**	NC	0.992		

**LVI (present vs absent)**	0.39 (0.08–1.79)	0.230	-	-

**PNI (present vs absent)**	0.43 (0.10–1.82)	0.255	-	-

**Ca19-9 (continuous variable)**	1.00 (1.00–1.00)	**0.100**	1.00 (1.00–1.00)	0.093

**CEA (continuous variable)**	1.01 (0.99–1.04)	**0.168**	1.01 (0.98–1.04)	0.388

**Capecitabine**				
**Gemcitabine-capecitabine**	1.21 (0.22–6.67)	0.823	-	-
**Cisplatin-gemcitabine**	1.25 (0.11–14.01)	0.851		
**Gemcitabine**	3.20 (0.43–23.61)	0.253		
**XELOX**	NC	0.990		
**(All compared to no adjuvant treatment)**	3.94 (0.34–45.64)	0.272		

**Adjuvant chemotherapy cycle (continuous variable)**	0.66 (0.45–0.96)	**0.032**	**0.63 (0.39–1.00)**	**0.050**

**Dose reduction (present vs. absent)**	0.87 (0.20–3.64)	0.853	-	-

**Abbreviations**. ECOG PS: Eastern Cooperative Oncology Group performance score; LVI: lymphovascular invasion; PNI: perineural invasion, Ca19-9: carbohydrate antigen 19-9, CEA: carcinoembryonic antigen XELOX: capecitabine and oxaliplatin.

**Table 4:** Characteristics of patients with advanced biliary tract cancer.

*Variable*	*N=90*

**Sex, n (%)**	
**Male**	**49 (54.4)**
**Female**	**41 (45.6)**

**Age at diagnosis, median (min-max)**	**67 (39–89)**

**ECOG PS, n (%)**	
**0**	**13 (14.4)**
**1**	**49 (54.4)**
**2**	**13 (14.4)**
**3**	**11 (12.2)**
**4**	**3 (3.3)**
**Unknown**	**1 (1.1)**

**Comorbidities, n (%)**	
**Hypertension**	**39 (43.3)**
**Diabetes mellitus**	**33 (36.7)**
**Cardiac disease**	**17 (18.9)**
**Hypothyroidism**	**9 (10)**
**Viral hepatitis**	**10 (11.1)**
**Cirrhosis**	**6 (6.7)**

**Smoking history, n (%)**	**40 (44.4)**

**Regular alcohol consumption, n (%)**	**10 (11.1)**

**Tumor localization, n (%)**	
**Intrahepatic**	**46 (51.1)**
**Perihilar**	**18 (20)**
**Distal**	**10 (11.1)**
**Gallbladder**	**16 (17.8)**

**Initially advanced disease, n (%)**	**62 (68.9)**
**Recurrent disease**	**28 (31.1)**
- **Previous adjuvant treatment**	**14/28 (50)**

**Metastatic sites, n (%)**	
**Liver**	**76 (84.4)**
**Lymph nodes**	**51 (56.7)**
**Peritoneal**	**21 (23.3)**
**Lung**	**24 (26.7)**
**Bone**	**12 (13.3)**

**Metastatic site number, median (min-max)**	**2 (1–4)**

**Molecular testing, n (%)** **MSI (high-stable-unknown)** **PD-L1 (positive-negative-unknown)** **HER2 (positive-negative-unknown)** **NGS panel (performed-not performed)** **Genes that have mutations in NGS:** ***KRAS*** ** (n=2), ** ***NRAS, KIT, RET, TP53*** ** (n=8), ** ***POLE, PTEN*** ** (n=2), ** ***CTNBB1, MSH3, BRAF*** ** (n=2), ** ***HER2, TERT, NF1, BRCA2*** ** (n=2), ** ***BRCA1, FGFR2, CDKN2A, SDHA***	**25/4/61 (27.8/4.4/ 67.8)** **8/10/72 (8.9/11.1/80)** **7/1/82 (7.8/1.1/91.1)** **24/66 (26.7/73.3)**

**Ca19-9, U/mL, median (min-max)**	**105.0 (0.8–19960.0)**

**CEA, ng/mL, median (min-max)**	**3.0 (0.2–1147.2)**

**Abbreviations**: ECOG PS: Eastern Cooperative Oncology Group performance score; MSI: microsatellite instability; PD-L1: programmed death ligand 1, HER2: human epidermal growth factor receptor 2; NGS: next-generation sequencing, Ca19-9: carbohydrate antigen 19-9, CEA: carcinoembryonic antigen.

**Table 5:** First-line chemotherapy eligibility, patterns, and tolerance in patients with advanced biliary tract cancer.

*Variable*	*n=90*

**First-line chemotherapy received, n (%)**	**75/90 (83.3)**
**Cisplatin-Gemcitabine**	**28 (31.1)**
**Carboplatin-Gemcitabine**	**3 (3.3)**
**FOLFOX/XELOX**	**24 (26.7)**
**FOLFIRINOX**	**4 (4.4)**
**Gemcitabine-Capecitabine**	**2 (2.2)**
**Gemcitabine-Oxaliplatine**	**1 (1.1)**
**Gemcitabine**	**1 (1.1)**
**Capecitabine**	**4 (4.4)**
**Local ablative treatment**	**7 (7.8)**
**Nivolumab**	**1 (1.1)**

**Dose reduction required, n (%)**	**53/75 (70.7)**

**First-line treatment cycle, median (min-max)**	**3 (1–18)**

**Grade 3/4 adverse events, n (%)**	**39/75 (52)**
Myelosuppression-cytopenia (n=10)	
Infection (n=12)	
Nause-vomiting (n=9)	
Diarrhea (n=2)	
Pulmonary embolism (n=2)	
Deep-vein thrombosis (n=1)	
Hand-foot syndrome (n=1)	
Hypersensitivity reaction (n=2)	
Fatigue (n=4)	
Acute kidney injury (n=4)	
Angina (n=1)	
Peripheral neuropathy (n=2)	
Hepatotoxicity (n=2)	

**Response to first-line treatment, n (%)**	
**Complete**	**4/75 (5.3)**
**Partial**	**18/75 (24.0)**
**Stable**	**18/75 (24.0)**
**Progressive**	**35/75 (46.7)**

**Able to receive second-line treatment, n (%)**	**33/75 (44.0)** **[33/90 (36.7)]**

**Abbreviations**. FOLFOX: fluorouracil, leucovorin, and oxaliplatin; XELOX: capecitabine and oxaliplatin, FOLFIRINOX: fluorouracil, leucovorin, irinotecan, and oxaliplatin.

**Table 6:** Univariable and multivariable Cox regression analysis for overall survival (OS) in patients with advanced biliary tract cancer who received first-line chemotherapy.

	Univariable		Multivariable	
	
Variable	HR (95% CI)	p	HR (95% CI)	p

**Sex (Male vs. female)**	0.69 (0.37–1.25)	0.227	-	-

**Age (continuous variable)**	1.02 (0.99–1.05)	**0.107**	0.99 (0.94–1.05)	0.895

**ECOG PS**				
**1 vs 0**	3.17 (1.09–9.13)	**0.033**	2.40 (0.32–17.76)	0.390
**2 vs 0**	4.65 (1.31–16.53)	**0.017**	0.89 (0.08–9.55)	0.924
**3 vs 0**	4.89 (1.06–22.49)	**0.041**	1.00 (0.03–29.30)	0.998

**Smoking history (present vs absent)**	0.77 (0.41–1.43)	0.414	-	-

**Comorbidities (present vs. absent)**				
**Hypertension**	1.44 (0.78–2.66)	0.244	-	-
**Diabetes mellitus**	1.26 (0.67–2.34)	0.461		
**Cardiac disease**	1.18 (0.52–2.67)	0.686		
**Hypothyroidism**	0.66 (0.23–1.88)	0.444		
**Viral hepatitis**	0.74 (0.26–2.09)	0.576		
**Cirrhosis**	0.68 (0.16–2.83)	0.598		

**Alcohol use (present vs absent)**	1.40 (0.63–3.10)	0.396	-	-

**Localization**				
**Perihilar vs intrahepatic**	1.11 (0.50–2.43)	0.793	0.72 (0.23–2.21)	0.568
**Distal vs intrahepatic**	1.20 (0.50–2.84)	0.679	0.57 (0.11–2.78)	0.488
**Gallbladder vs intrahepatic**	2.55 (1.10–5.90)	**0.028**	1.89 (0.52–6.86)	0.333

**Presentation (de novo vs recurrent)**	1.86 (0.95–3.62)	**0.067**	2.81 (0.90–8.79)	0.075

**Metastatic sites (present vs. absent)**				
**Liver**	0.63 (0.28–1.44)	0.283	-	-
**Lymph node**	1.57 (0.85–2.89)	**0.142**	1.58 (0.59–4.19)	0.358
**Peritoneal**	1.55 (0.80–2.99)	**0.193**	2.53 (0.88–7.27)	0.083
**Lung**	0.75 (0.37–1.50)	0.421	-	-
**Bone**	1.09 (0.51–2.36)	0.812	-	-

**Ca19-9 (continuous variable)**	1.00 (1.00–1.00)	0.980	-	-

**CEA (continuous variable)**	1.00 (0.99–1.00)	0.263	-	-

**Cisplatin-Gemcitabine**	0.13 (0.04–0.43)	**0.000**	**0.10 (0.01–0.58)**	**0.010**
**Carboplatin-Gemcitabine**	0.67 (0.12–3.76)	0.658	-	-
**FOLFOX/XELOX**	0.45 (0.15–1.35)	**0.158**	0.54 (0.11–2.51)	0.436
**FOLFIRINOX**	0.18 (0.03–1.03)	**0.054**	0.11 (0.00–1.51)	0.099
**Gemcitabine-Capecitabine** **(All compared to capecitabine)**	0.64 (0.11–3.57)	0.620	-	-

**First-line chemotherapy cycle (continuous variable)**	0.72 (0.61–0.86)	**0.000**	**0.58 (0.45–0.76)**	**0.000**

**Dose reduction (present vs. absent)**	3.29 (1.56–6.94)	**0.002**	1.74 (0.55–5.50)	0.341

**Second-line chemotherapy (received vs not received)**	0.32 (0.17–0.59)	**0.000**	**0.28 (0.11–0.68)**	**0.005**

**Abbreviations**. ECOG PS: Eastern Cooperative Oncology Group performance score Ca19-9: carbohydrate antigen 19-9, CEA: carcinoembryonic antigen FOLFOX: fluorouracil, leucovorin, and oxaliplatin; XELOX: capecitabine and oxaliplatin, FOLFIRINOX: fluorouracil, leucovorin, irinotecan, and oxaliplatin.

**Table 7:** Sensitivity analysis for treatment discontinuation due to toxicity: Univariable and multivariable Cox regression analysis for overall survival (OS) in patients with advanced biliary tract cancer who received first-line chemotherapy and did not experience early progression.

	Univariable		Multivariable	

Variable	HR (95% CI)	P	HR (95% CI)	P

**Sex (male vs. female)**	0.64 (0.34–1.20)	**0.174**	**3.61 (1.12–11.59)**	**0.034**

**Age (continuous variable)**	1.03 (1.00–1.06)	**0.046**	0.98 (0.93–1.04)	0.647

**ECOG PS**				
**1 vs 0**	4.24 (1.27–14.18)	**0.019**	7.01 (0.96–51.20)	0.055
**2 vs 0**	5.63 (1.35–23.42)	**0.017**	3.05 (0.31–30.07)	0.338
**3 vs 0**	6.69 (1.31–34.17)	**0.022**	25.85 (1.31–50.47)	0.052

**Smoking history (present vs absent)**	0.70 (0.36–1.34)	0.283	-	-

**Comorbidities (present vs. absent)**			-	-
**Hypertension**	1.49 (0.78–2.84)	0.220		
**Diabetes mellitus**	1.26 (0.65–2.43)	0.486		
**Cardiac disease**	1.27 (0.56–2.91)	0.560		
**Hypothyroidism**	0.69 (0.24–1.99)	0.502		
**Viral hepatitis**	0.78 (0.27–2.21)	0.646		
**Cirrhosis**	0.71 (0.17–2.98)	0.645		

**Alcohol use (present vs absent)**	1.44 (0.59–3.51)	0.414	-	-

**Localization**				
**Perihilar vs intrahepatic**	1.24 (0.55–2.78)	0.594	0.79 (0.24–2.53)	0.692
**Distal vs intrahepatic**	1.24 (0.48–3.15)	0.650	0.18 (0.03–1.15)	0.071
**Gallbladder vs intrahepatic**	2.92 (1.23–6.90)	**0.015**	1.48 (0.51–4.27)	0.463

**Presentation (de novo vs recurrent)**	1.74 (0.88–3.44)	**0.107**	**3.24 (1.11–9.45)**	**0.031**

**Metastatic sites (present vs. absent)**				
**Liver**	0.59 (0.25–1.35)	0.216	-	-
**Lymph node**	1.46 (0.78–2.73)	0.234	-	-
**Peritoneal**	1.71 (0.87–3.38)	**0.117**	1.51 (0.56–4.09)	0.414
**Lung**	0.79 (0.38–1.62)	0.526	-	-
**Bone**	1.16 (0.51–2.64)	0.711	-	-

**Ca19-9 (continuous variable)**	1.00 (1.00–1.00)	0.967	-	-

**CEA (continuous variable)**	1.01 (0.99–1.00)	0.226	-	-

**Cisplatin-Gemcitabine**				
**Carboplatin-Gemcitabine**	0.11 (0.03–0.37)	**0.000**	**0.16 (0.02–0.97)**	**0.047**
**FOLFOX/XELOX**	0.67 (0.12–3.75)	0.654	-	-
**FOLFIRINOX**	0.43 (0.14–1.30)	**0.138**	1.20 (0.21–6.88)	0.873
**Gemcitabine-Capecitabine**	0.10 (0.01–0.99)	**0.050**	0.28 (0.01–4.73)	0.378
**(All compared to capecitabine)**	0.67 (0.12–3.75)	0.654	-	-

**Treatment discontinuation due to toxicity (present vs absent)**	2.97 (1.58–5.65)	**0.001**	**3.26 (1.34–7.93)**	**0.009**

**Dose reduction (present vs. absent)**	3.35 (1.52–7.38)	**0.003**	0.91 (0.28–2.96)	0.878

**Second-line chemotherapy (Received vs not received)**	0.32 (0.16–0.61)	**0.001**	**0.29 (0.12–0.69)**	**0.005**

**Abbreviations**. ECOG PS: Eastern Cooperative Oncology Group performance score Ca19-9: carbohydrate antigen 19-9, CEA: carcinoembryonic antigen FOLFOX: fluorouracil, leucovorin, and oxaliplatin; XELOX: capecitabine and oxaliplatin, FOLFIRINOX: fluorouracil, leucovorin, irinotecan, and oxaliplatin.
